# Design, Synthesis, and Characterization of Novel Pyrazole Cross-Linked Chitosan Derivatives Modified with Zinc Oxide Nanoparticles for Boosting Their Anticancer Activity

**DOI:** 10.3390/polym17081061

**Published:** 2025-04-15

**Authors:** Hanan D. Almutairi, Marwa Abdel-Motaal, Marwa Sharaky, Nadia A. Mohamed

**Affiliations:** 1 Department of Chemistry, College of Science, Qassim University, Buraidah 51452, Saudi Arabia; ma.mohammed@qu.edu.sa; 2 Pharmacology Unit, Cancer Biology Department, National Cancer Institute (NCI), Cairo University, Cairo 11796, Egypt; marwa.sharaky@nci.cu.edu.eg

**Keywords:** chitosan, pyrazole, synthesis, ZnO nanoparticles, anticancer activity, cytotoxicity

## Abstract

A new series of chitosan-based pyrazole derivatives was successfully prepared via crosslinking chitosan using either malonopyrazole (MPy-Cs) or thiopyrazole (TPy-Cs). Three derivatives of TPy-Cs were produced based on their content of TPy, namely TPy-Cs1, TPy-Cs2, and TPy-Cs3 of crosslinking degrees of 71, 48, and 29%, respectively. Further, various weight ratios of ZnO nanoparticles were loaded into some of these derivatives to obtain the corresponding ZnONP bio-composites. FTIR, XRD, SEM, and TEM techniques were employed to emphasize the chemical, internal, and morphological structure of these derivatives. Although MPy-Cs derivatives did not show any activity against all the examined cancer cell lines, TPy-Cs derivatives exhibited an appreciable anticancer activity which greatly improved with increasing their TPy content, i.e., from TPy-Cs3 to TPy-Cs1. The TPy-Cs1 displayed IC_50_ (14.4 μg/mL) against the HN9 cell line that was comparable to the Doxorubicin (DOX) standard drug (12.6 μg/mL). Among all the prepared composites, TPy-Cs3/ZnONPs-5% was the most potent anticancer candidate against all the tested cancer cell lines, although it does not exceed the anticancer activity of DOX. Tpy-Cs2 and its ZnONP composites were safe on normal human skin fibroblast (HSF) cell lines. Thus, the inclusion of both TPy and ZnONPs into the chitosan matrix fostered its anticancer efficiency.

## 1. Introduction

Cancer is a leading global cause of death, resulting from abnormal cell proliferation that spreads throughout the body. Chemotherapy drugs play a crucial role in combating this deadly disease [1]. To enhance survival rates and decrease tumor size, numerous cancer patients depend on the use of chemotherapeutic medications [2]. While chemotherapeutic drugs enhance the effectiveness of cancer treatment, certain patients may endure drug-induced side effects [3].

The high-dose requirement, poor bioavailability, low selectivity index, development of drug resistance, and non-specific interactions are major drawbacks of chemotherapeutic drugs. Therefore, there is an urgent need to develop a suitable drug delivery system to reduce the therapeutic dose or frequency, and thereby minimize the toxic effects of the anticancer drugs [4,5].

Biopolymers are utilized as carriers in delivering active pharmaceutical ingredients. They play a crucial role in developing various delivery systems, such as hydrogels, micelles, tablets, capsules, and particulate systems (nanoparticles, beads, and micro) [6,7,8]. As a carrier, the biopolymer must be non-toxic, biodegradable, and biocompatible. The latter two properties help to remove the carrier after drug administration [9,10]. Some biopolymers used in drug delivery systems include cellulose, alginate, gellan gum, pectin, gum arabic, guar gum, starch, gelatin, chondroitin sulfate, and hyaluronic acid [11]. Among these, chitosan is one of the most widely used biopolymers in the pharmaceutical industry [12,13,14].

Chitosan is a biocompatible and biodegradable polymer that is derived from chitin [15]. Chitosan shows various biochemical activities such as antiproliferative and antimicrobial activity, immune activation, cholesterol level-lowering activities, production of phytoalexin-eliciting activities, antihypertensive action(s), and neuroprotective, wound-healing, and antiulcer activities [16,17]. Chitosan is frequently utilized as a delivery vehicle for nucleic acids, chemotherapeutic drugs, or anticancer compounds due to its biocompatibility. Additionally, chemically modified chitosan derivatives can be used as antiproliferative agents, either on their own or in conjunction with other anticancer agents [18,19].

Chitosan can cause programmed death of cancer cells, often by activating caspases and other apoptotic pathways [20]. It also helps to inhibit tumor growth and metastasis by reducing inflammation [21]. Chitosan increases the activity of immune cells like macrophages and natural killer (NK) cells, improving the ability of the body to target cancer cells [22]. The biocompatibility of the chitosan enables it to be used as a carrier for anticancer drugs, increasing efficacy while reducing side effects [23]. It also inhibits the formation of new blood vessels, which tumors require to grow and spread [24], and may influence metabolic pathways in cancer cells, disrupting their energy production and viability [25].

Pyrazoles are five-membered heterocycles that form a class of compounds particularly useful in organic synthesis. They are one of the most studied groups of compounds within the azole family. A wide variety of synthesis methods and synthetic analogues have been reported over the years. The presence of the pyrazole nucleus in different structures leads to diverse applications in areas such as technology, medicine, and agriculture. They are described as inhibitors of protein glycation, and have been found to possess antibacterial, antifungal, anticancer, antidepressant, anti-inflammatory, anti-tuberculosis, antioxidant, and antiviral properties [26,27].

Nowadays, pyrazole systems have garnered increased attention as biomolecules due to their intriguing pharmacological properties. This heterocycle can be found in numerous well-established drugs across diverse therapeutic categories [28,29]. Pyrazole derivatives frequently inhibit the proliferation of cancer cells. They can disrupt key signaling pathways that control cell cycle progression, resulting in apoptosis (programmed cell death). Many pyrazole compounds inhibit specific enzymes involved in cancer cell metabolism. For example, some pyrazole derivatives have been shown to inhibit cyclooxygenase (COX) enzymes, which are involved in tumor growth and progression. Pyrazoles can activate apoptotic pathways in cancer cells by regulating the expression of apoptosis-related proteins such as Bcl-2 family proteins and caspases. Certain pyrazole derivatives may inhibit angiogenesis, the formation of new blood vessels required for tumor growth. This may involve inhibiting the vascular endothelial growth factor (VEGF) signaling pathway. Pyrazoles can influence key signaling pathways, including the PI3K/Akt and MAPK pathways, which are frequently dysregulated in cancer. Some pyrazoles can cause oxidative stress in cancer cells, resulting in their death. The increased reactive oxygen species (ROS) levels can damage cellular components, accelerating apoptosis [30,31].

Zink oxide nanoparticles (ZnONPs) can trigger apoptosis in cancer cells via intrinsic pathways. This process involves mitochondrial dysfunction, which results in the release of cytochrome C into the cytosol, activating caspases and initiating apoptotic cascades [32]. ZnONPs have been shown to produce ROS within cancer cells, which cause oxidative stress. Increased ROS levels can cause cellular damage, disrupt redox balance, and activate apoptotic pathways [33,34]. ZnONPs upregulate cyclin-dependent kinase inhibitors, which can result in cell cycle arrest in the G1, S, or G2/M phases. This stops cancer cells from growing and can make other therapies more successful [35]. By preventing the production of blood vessels, ZnONPs may have anti-angiogenic effects by blocking vascular endothelial growth factors. This restricts the access of the tumors to oxygen and nutrition, which stops them from growing [36]. According to some research, ZnONPs have the ability to specifically target cancer stem cells, which are essential for tumor dissemination and recurrence. ZnONPs can enhance overall therapy results by decreasing the population of cancer cells [37]. ZnONPs have the ability to improve the effectiveness of other therapeutic treatments, including radiation and chemotherapy. Better treatment results and fewer negative effects may result from this synergistic impact [38].

Accordingly, in this work and for the first time, the anticancer activity of chitosan was boosted by chemical crosslinking via its primary -NH_2_ groups using the bioactive bis-pyrazole moieties. This was achieved by preparing a series of new pyrazole cross-linked chitosan derivatives via reacting chitosan with a bis-pyrazole linked with either a malonic linkage (MPy-Cs) or a thiocarbonyl group (TPy-Cs). The incorporation of nitrogen-rich pyrazole nuclei, substituted with amino, ester, and thiocarbonyl groups, as cross-linkages between the chitosan chains, leads to enhancing the interactions between their positively charged sites and the negatively charged cancer cell membranes. Also, some new ZnONPs/pyrazole cross-linked chitosan composites were prepared by dispersing two different ZnONP concentrations of 3 and 5% based on the weight of MPy-Cs and TPy-Cs derivatives, to improve their anticancer activity. Thus, the amalgamation of bis-pyrazole, ZnONPs, and chitosan in the same structure is considered to be a good strategy to accomplish adequate systems for competing with the traditional anticancer agents. The structure of the prepared derivatives and their ZnONPs/composites was characterized using FTIR, XRD, SEM and TEM. Their efficacy against three different types of cancer cells (human colorectal carcinoma cells (HCT_116_), human skin carcinoma cells (A375), and human tongue carcinoma cells (HN9)) was investigated. Their cytotoxicity against human skin fibroblasts HSF was also evaluated.

## 2. Materials and Methods

### 2.1. Materials

Chitosan (Cs, 1.0–3.0 × 10^5^ g mol^−1^ molecular weight, 98% degree of deacetylation) was purchased from Acros Organics (Morris Plains, NJ, USA). Carbon disulfide (99.9%), dimethyl sulfate (99%), dimethyl sulfoxide anhydrous (DMSO, 99.9%), and thiocarbohydrazide (98%) were provided by Sigma-Aldrich (Munich, Germany). Ethyl cyanoacetate (98%) was supplied by Loba Chemie (Mumbai, India). Potassium hydroxide pellets (98%) were obtained from Pan-Reac. AppliChem-ITW Reagent (Darmstadt, Germany). Dimethylformamide and methanol were purchased from Fisher Scientific UK Ltd. (Leicestershire, UK). Acetone was provided by NV (Zedelgem, Belgium). Zinc oxide nanoparticles (ZnONPs) (product number: NCZ4701, CAS number: 1314-13-2, purity > 99.99%, particle size: 20 nm, form: nanopowder) were supplied by Nanochemazodne Inc. (Leduc, AB, Canada). Malonohydrazide and ethyl 2-cyano-3,3-bis(methylthio)acrylate were prepared according to the methods previously described [39,40]. Three human cancer cell lines included human colorectal carcinoma cells (HCT_116_), human skin carcinoma cells (A375), and human tongue carcinoma cells (HN9), in addition to normal human skin fibroblasts (normal HSF) which were obtained from the American Type Culture Collection (ATCC, Manassas, VA, USA). They were maintained at the National Cancer Institute (NCI), Cairo University, Cairo, Egypt, in DMEM media containing 10% fetal bovine serum and 1% penicillin-streptomycin, and incubated in 5% CO_2_ in a humidified atmosphere at 37 °C.

### 2.2. Methods

#### 2.2.1. Synthesis of Novel Malonopyrazole Cross-Linked Chitosan (MPy-Cs) Derivative

**Figure 1:**
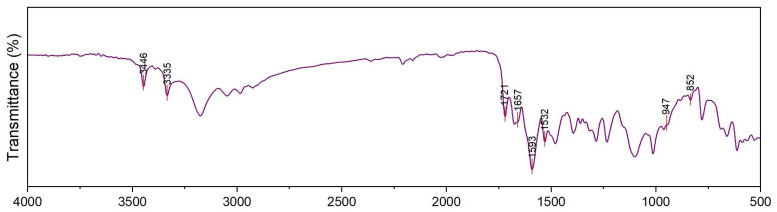
FTIR spectrum of MPy.

**Figure 2:**
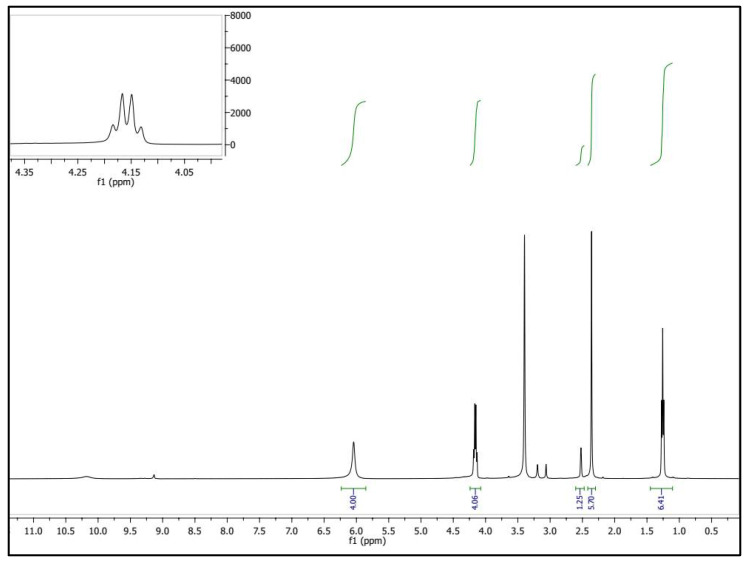
^1^H-NMR spectrum of MPy.

**Figure 3:**
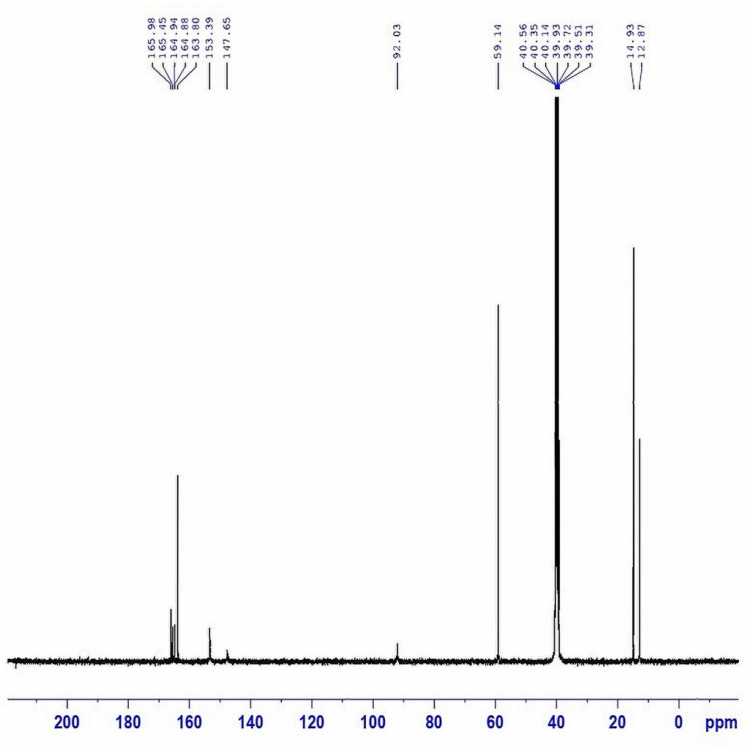
^13^C-NMR spectrum of MPy.

**Scheme 1:**
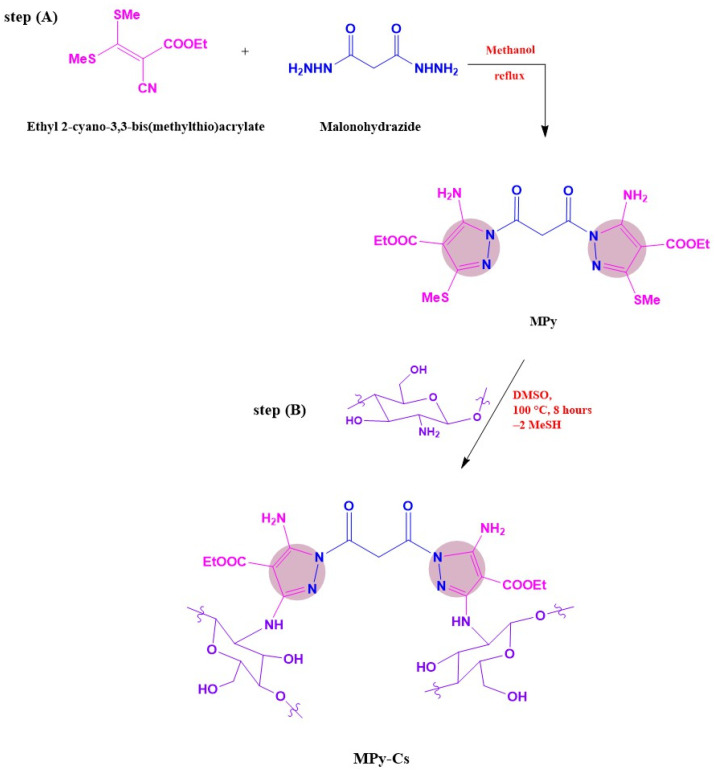
Synthesis of novel MPy-Cs derivative: (**A**) synthesis of MPy; (**B**) synthesis of MPy-Cs.

Step A: A mixture of malonohydrazide (0.1 mmol) and ethyl 2-cyano-3,3-bis(methylthio)acrylate (0.2 mmol) was refluxed in 20 mL of methanol for 2 h. The formed precipitate was filtered off, dried, and crystallized from methanol to afford the pure bis-pyrazole product (MPy, Scheme 1A) as faint brown crystals (yield = 85%, mp = 180–182 °C). IR (KBr) *ν*_max_ (cm^−1^): 3446 and 3335 (NH_2_), 1721 (C=O ester), 1657 (C=O amide and C=N in the pyrazole rings), 1593 and 1532 (C=C in the pyrazole rings), 947 and 852 (C-S) as shown in Figure 1. ^1^H-NMR (DMSO-*d*6) *d* (ppm) (Figure 2): 1.26 (t, *J* = 7.03 Hz, 6H, 2CH_3_ ester), 2.36 (s, 6H, 2SCH_3_), 3.3 (s, 2H, CH_2_), 4.13 (q, *J* = 7.01 Hz, 4H, 2CH_2_ of ester), 6.04 (s, 4H, 2NH_2_) and ^13^C-NMR (DMSO-*d*6) δ (ppm) (Figure 3): 11.87(-CH_3_, ester group), 14.93 (-CH_3_, -SMe), 59.14 (-CH_2_, ester), 92.03 (-CO-CH_2_-CO-, 147.65, 153.39, 163.8, 164.88, 164.94 (-C-SMe), 165.45, 165.98 (2 -C=O). MS (70 eV, EI, %), m/z = 487 (M+, 19), Anal. Calcd.: %C, 43.40; %H, 4.71; %N, 17.86; %S, 13.63 (470.52): Found: %C, 43.38; %H, 4.69; %N, 17.85; %S, 13.66.

Step B: MPy (0.1 mol) was added to chitosan (0.2 mol, already swelled in 50 mL DMSO) and stirred at 100 °C for 8 h, and then at RT for 12 h, and thereafter poured into a beaker containing 400 mL acetone with gentle stirring. The reaction mixture was cooled at 4 °C for 4 h, and the formed MPy-Cs derivative (Scheme 1B) was filtered. Exhaustive extraction using a Soxhlet for 10 h of the MPy-Cs with methanol allowed for its purification from the unreacted MPy. The MPy-Cs after extraction was washed well with hot methanol and dried in an air-circulating oven at 60 °C until reaching a constant weight.

#### 2.2.2. Synthesis of Novel Thiopyrazole Cross-Linked Chitosan (TPy-Cs) Derivatives

**Scheme 2:**
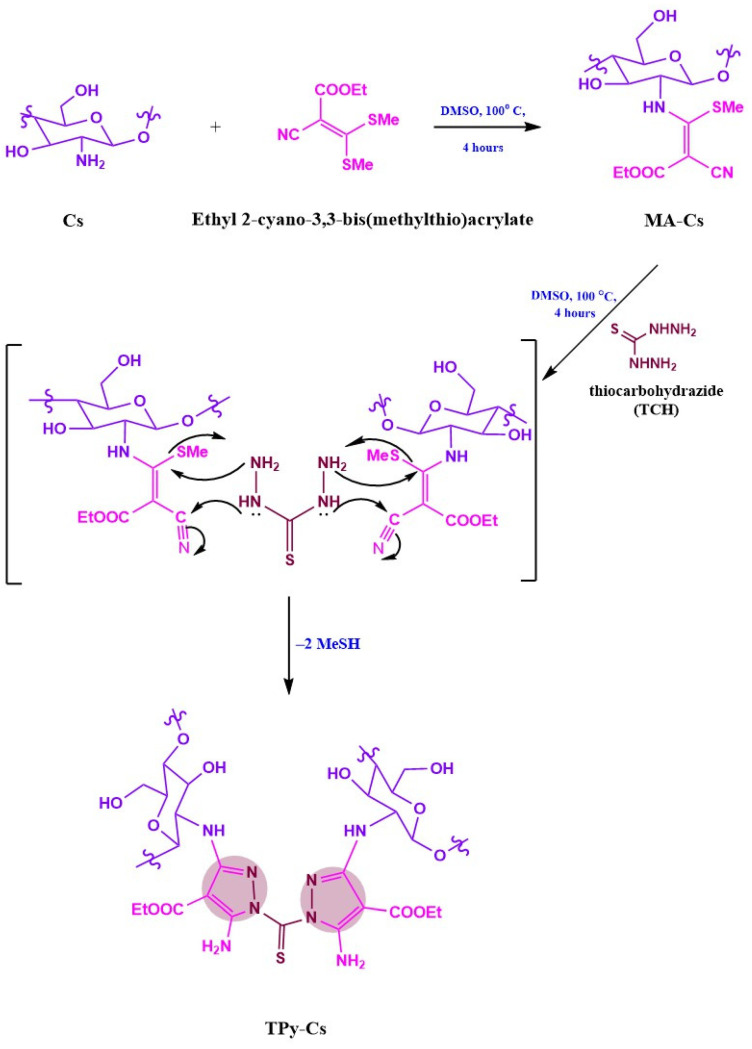
Synthesis of novel TPy-Cs derivatives.

TPy-Cs derivatives were prepared by adding ethyl 2-cyano-3,3-bis(methylthio)acrylate (0.2 mol) to the suspended chitosan (0.2 mol) in DMSO (30 mL). The mixture was stirred at 100 °C for 4 h, and then at RT for 12 h, obtaining MA-Cs (Scheme 2). After that, three different predetermined quantities of thiocarbohydrazide (TCH, Scheme 2 and Table 1) were added in situ and stirred at 100 °C for 4 h then at RT for 12 h. The reaction mixtures were poured into a beaker containing 400 mL acetone with stirring. After cooling at 4 °C for 4 h, the formed products (TPy-Cs derivatives) were filtered off. The unreacted ethyl 2-cyano-3,3-bis(methylthio)acrylate and thiocarbohydrazide were extracted from the TPy-Cs derivatives with methanol using a Soxhlet apparatus for 10 h. The TPy-Cs derivatives after extraction were washed well with hot methanol and dried in an air-circulating oven at 60 °C until reaching a constant weight. The reactant molar ratio was listed in Table 1 to obtain three novel thiopyrazole cross-linked chitosan (TPy-Cs) derivatives denoted as TPy-Cs1, TPy-Cs2, and TPy-Cs3 of decreasing the TPy moiety content, respectively.

#### 2.2.3. Synthesis of TPy-Cs/ZnONPs and MPy-Cs/ZnONP Composites

**Scheme 3:**
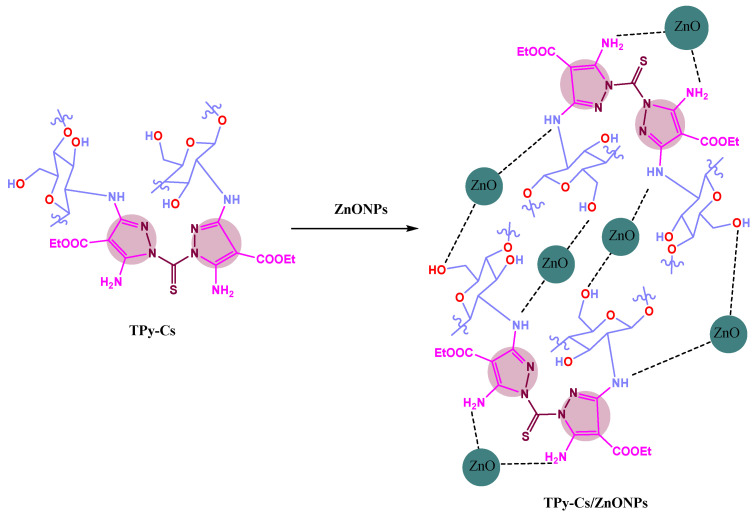
Presumptive presentation of the interaction between TPy-Cs and ZnONPs in their composites.

Using a mortar, two different precalculated quantities of ZnONPs were crushed separately with a fixed weight of either the Soxhlet-purified TPy-Cs2, TPy-Cs3, or MPy-Cs (0.25 g), then stirred in 10 mL of acetone for 24 h at room temperature. The ZnONP bio-composites were filtered and dried at 55 °C. The used ZnONP quantities were 3 and 5% based on the weight of the TPy-Cs and MPy-Cs derivatives. Thus, six composites, designated as TPy-Cs2/ZnONPs-3%, TPy-Cs2/ZnONPs-5%, TPy-Cs3/ZnONPs-3%, and TPy-Cs3/ZnONPs-5%, MPy-Cs/ZnONPs-3% and MPy-Cs/ZnONPs-5%, were produced. The interaction of TPy-Cs with ZnONPs in their composites, as a representative example, is shown in Scheme 3.

### 2.3. Measurements

#### 2.3.1. FTIR Spectroscopy

FTIR spectroscopy measurements of pyrazole cross-linked chitosan derivatives and their ZnONP composites were recorded on Agilent Technologies FTIR Spectrometer (Cary 600 Series, Santa Clara, CA, USA) in the wavenumber range from 4000 to 400 cm^−1^.

#### 2.3.2. X-Ray Diffractometry (XRD)

X-ray diffractometer (Joel JDX-8030, Akishima, Japan) was utilized to investigate the internal structure of the pyrazole cross-linked chitosan derivatives and their ZnONP composites at a diffraction angle (2θ) throughout a range of 5 to 90°, with a 5° min^−1^ speed.

#### 2.3.3. Scanning Electron Microscopy (SEM)

The surface morphology of the gold-coated pyrazole cross-linked chitosan derivatives and their ZnONP composites was studied using an ultra-high-resolution Schottky Field Emission SEM device (JEOL 7610F, Tokyo, Japan).

#### 2.3.4. Transmission Electron Microscopy (TEM)

The inspection of the morphology of the suspended solution of the TPy-Cs2/ZnONPs-5% was carried out using an advanced high-resolution Field Emission Transmission Electron Microscope (HR-JEOL, JEM-2100F, Tokyo, Japan).

#### 2.3.5. Anticancer and Cytotoxicity Assay

The activity of MPy-Cs, TPy-Cs derivatives and their ZnONP composites beside the Doxorubicin (DOX, as a reference drug) against HCT_116_ (human colorectal carcinoma cells), A375 (human skin carcinoma cells) and HN9 (human tongue carcinoma cells), and the normal HSF (human skin fibroblasts) cell lines was evaluated by sulphorhodamine-B (SRB) assay [41]. In brief, the cells were seeded at a density concentration of 3 × 10^3^ cells/well in 96-well microtiter plates They were allowed to attach for 24 h before being incubated with the investigated derivatives and their nanocomposites. Consequently, cells were treated with doses 25, 50, 100, and 200 μg/mL of these samples solubilized in DMSO. Five wells were utilized for every concentration with continued incubation for 48 h. Dimethyl sulfoxide (DMSO) was used as the control medium (1% *v*/*v*) with no effect on the experiment. After terminating the incubation, the cells were fixed with 20% trichloroacetic acid and each well was colored with 0.4% SRB dye. Each well’s optical density (O.D.) was measured spectrophotometrically at 570 nm using an ELISA microplate reader (TECAN sunrise™, Männedorf, Switzerland). The mean survival percentage of the cells was calculated as follows: O.D. (treated cells)/O.D (control cells) × 100. The Half-maximal Inhibitory Concentration (IC_50_) value of each sample was calibrated from the graph of the dose–response curve for each concentration using Graph Pad Prizm software (version 8.2).

Further, in this work, we used the outgrowth technique to generate the primary culture of normal human skin fibroblasts (HSF) from young people’s skin explants. HSF cells were displayed in samples (up to 200 μg/mL) for 48 h.

## 3. Results and Discussion

### 3.1. Synthesis of the MPy-Cs, TPy-Cs Derivatives and Their ZnONP Composites

Novel pyrazole cross-linked chitosan derivatives were synthesized via a chemical modification of chitosan by incorporating a bis-pyrazole linked with either a malonic linkage (MPy-Cs, Scheme 1) or thiocarbonyl group (TPy-Cs, Scheme 2) to enhance its anticancer activity.

The synthesis of MPy-Cs included two steps: first is a condensation of ethyl 2-cyano-3,3-bis(methylthio)acrylate with malonohydrazide (2:1) in boiling methanol with stirring for 2 h, obtaining MPy (Scheme 1A, its structure was affirmed depending on spectral measurements). Secondly, the target MPy-Cs was achieved by heating MPy with chitosan (1:2) in DMSO at 100 °C for 8 h to condense the -SMe groups of the synthesized MPy with the amino groups of chitosan (Scheme 1B).

On the other hand, the synthesis of a novel series of TPy-Cs involved firstly the formation of the intermediate MA-Cs via treating equimolar amounts of chitosan with ethyl 2-cyano-3,3-bis(methylthio)acrylate in DMSO with stirring at 100 °C for 4 h (Scheme 2). Afterward, three different molar ratios of thiocarbohydrazide (TCH) were condensed with MA-Cs (1:2) in situ to afford a series of TPy-Cs derivatives denoted as TPy-Cs1, TPy-Cs2, and TPy-Cs3 (Scheme 2 and Table 1).

Further, some ZnO nanocomposites based on MPy-Cs, TPy-Cs2, and TPy-Cs3 were prepared using two different concentrations of ZnONPs of 3 and 5% (based on the weight of these derivatives), producing MPy-Cs/ZnONPs-3%, MPy-Cs/ZnONPs-5%, TPy-Cs2/ZnONPs-3%, TPy-Cs2/ZnONPs-5%, TPy-Cs3/ZnONPs-3%, and TPy-Cs3/ZnONPs-5%.

### 3.2. Characterization of MPy-Cs, TPy-Cs Derivatives and Their ZnONP Composites

#### 3.2.1. FTIR Analysis

**Figure 4:**
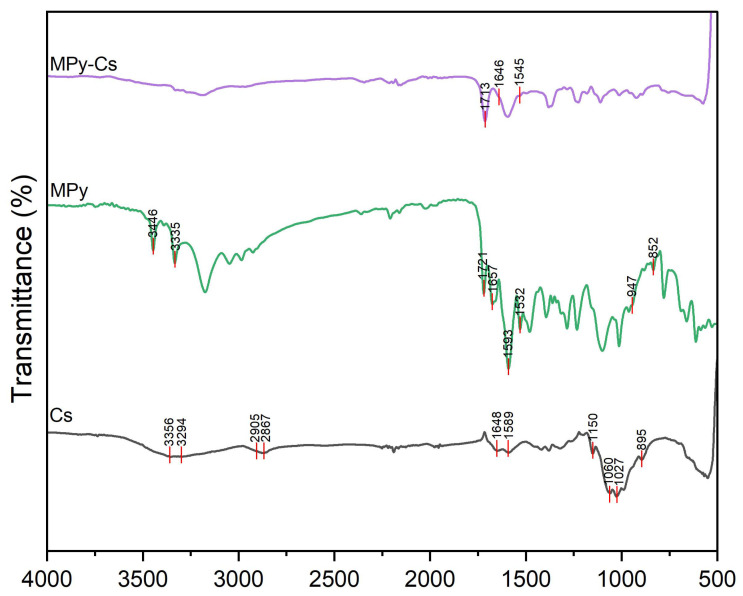
FTIR spectrum of MPy-Cs derivative.

FTIR spectrum of Cs (Figure 4) showed, between 3700 and 3000 cm^−1^, a very broad absorption peak, corresponding to the stretching vibration of the hydroxyl groups overlapping with that of -NH_2_ groups and their hydrogen bonds. In this wavenumber range, there is a doublet peak at 3356 and 3294 cm^−1^ related to the -NH_2_ groups. The symmetric stretching vibration peaks of the -CH and -CH_2_ groups in the moieties of pyranose appeared at 2905 and 2867 cm^−1^, respectively. Further, two weak peaks that appeared at 1648 and 1589 cm^−1^ are attributable to amide I and amide II, respectively, confirming the high Cs degree of deacetylation. The four absorption peaks that appeared at 1150, 1060, 1027, and 895 cm^−1^ confirmed the saccharide moieties of Cs [42].

In addition to the characteristic stretching vibration peaks of Cs (Figure 4) and MPy (Figure 1 and Figure 4), the FTIR spectrum of MPy-Cs (Figure 4) showed some additional distinguished peaks; at 1713 cm^−1^ related to the C=O of the ester, a broad peak ranged from 1646 to 1545 cm^−1^ attributed to the overlapped C=O of amide, C=C, and C=N of pyrazole rings.

**Figure 5:**
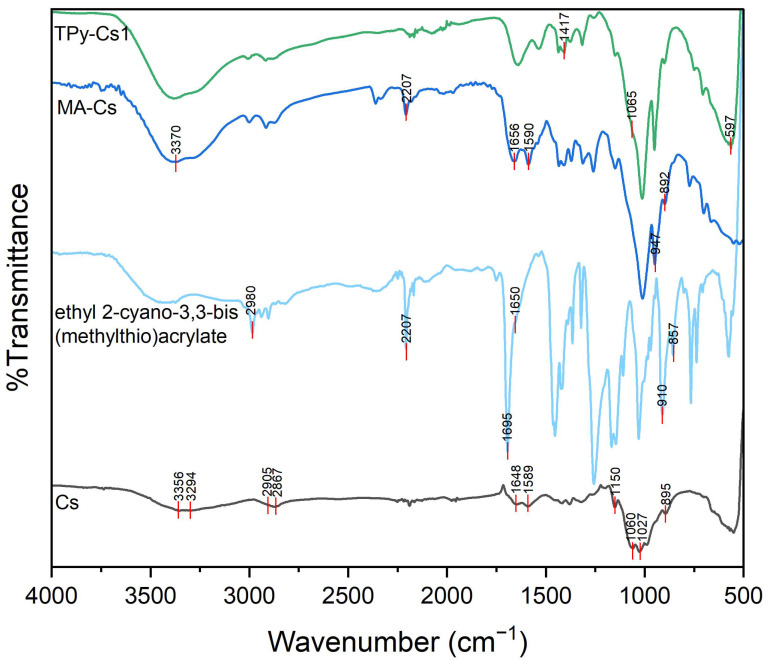
FTIR spectrum of TPy-Cs1 derivative.

FTIR spectrum of ethyl 2-cyano-3,3-bis(methylthio)acrylate (Figure 5, Scheme 2) showed some characteristic stretching vibration peaks at 2207 cm^−1^ assigned to CN group, at 1695 cm^−1^ corresponded to C=O of the ester, at 1650 cm^−1^ related to the C=C bond, at 2980 cm^−1^ indicating C-H bonds in the hydrocarbon chain, and at 910 and 857 cm^−1^ attributed to C-S groups [43].

FTIR spectrum of MA-Cs (Figure 5) showed stretching vibration peaks at 947 and 892 cm-1 ascribed to the C-S group, indicating the occurrence of the condensation reaction between Cs and ethyl 2-cyano-3,3-bis(methylthio)acrylate with the elimination of MeSH (Scheme 2). This is evidenced by the disappearance of the doublet peak of the primary amine groups of Cs at 3356 and 3294 cm^−1^, which was completely replaced by a peak at 3370 cm^−1^. In addition to the appearance of the absorption peaks, 2207, 1656 and 1590 cm^−1^ corresponded to the CN group, C=O group of the ester and C=C bonds, respectively.

FTIR spectra of TPy-Cs1 derivative (Figure 5) showed the disappearance of the peaks of C-S groups at 947 and 892 cm^−1^, indicating the condensation between the -SMe groups of MA-Cs and the -NH_2_ groups of the thiocabohydrazide (TCH) with the elimination of MeSH (Scheme 2). The peak corresponding to CN groups at 2207 cm^−1^ also disappeared. Further, the absorption peak of the C=S group at 1065 cm^−1^ overlapped with that of the C–O group, obtaining a broad, strong peak. The absorption peaks related to N–C–S groups appeared at 1417 and 597 cm^−1^ [44].

**Figure 6:**
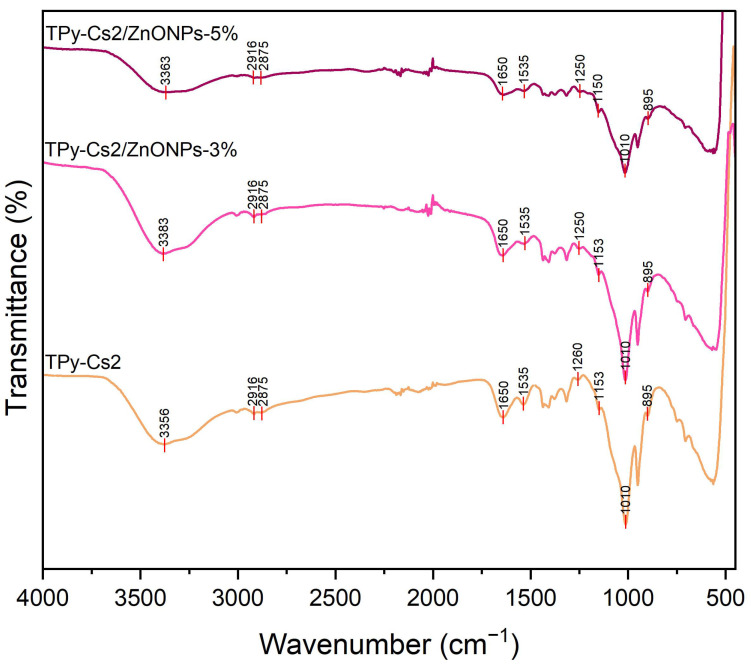
FTIR spectra of TPy-Cs2/ZnONP composites.

FTIR spectra of TPy-Cs2/ZnONPs-3% and TPy-Cs2/ZnONPs-5%, as representative examples for the ZnONP biocomposites, (Figure 6), showed absorption peaks comparable to those of their parent TPy-Cs2. The peaks at around the wavenumber of 566 cm^−1^, which corresponded to the expected N-ZnO and O-ZnO bonds in these biocomposites, seemed to overlap with those of their parent TPy-Cs2 [45].

#### 3.2.2. XRD Analysis

**Figure 7:**
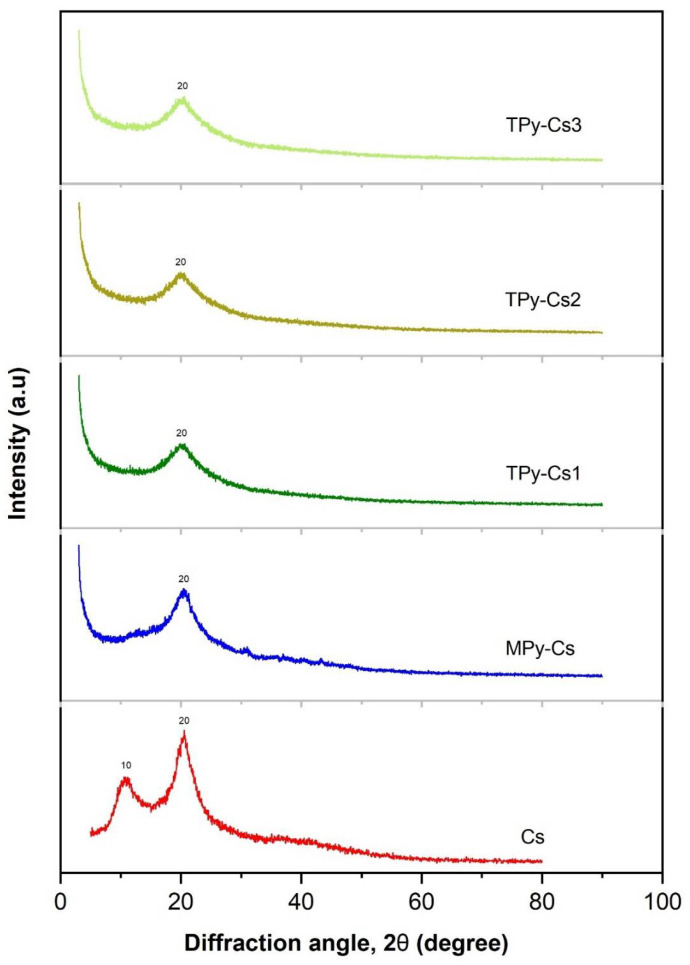
XRD patterns of MPy-Cs and TPy-Cs derivatives.

The virgin chitosan is distinguished by an inner structure involving amorphous and crystalline regions which manifested in its XRD pattern as two peaks near 2θ = 10° and 20°, respectively, as shown in Figure 7 [46]. This is ascribed to its capability to form a considerable number of hydrogen bonds due to its possession of a lot of hydroxyl and primary amine polar groups. Modification of chitosan with MPy and TPy leads to a remarkable reduction in its number of hydrogen bonds due to the consumption of its amino groups during the cross-linking process. This led to a separation of the chitosan chains away from each other, an increment in the amorphous region and a reduction in the crystalline fraction. This is shown from the evanescence of the peak at 2θ = 10° and a broadening and a notable lowering of the intensity of the peak at 2θ = 20° in XRD patterns of MPy-Cs, TPy-Cs1, TPy-Cs2, and TPy-Cs3 (Figure 7).

**Figure 8:**
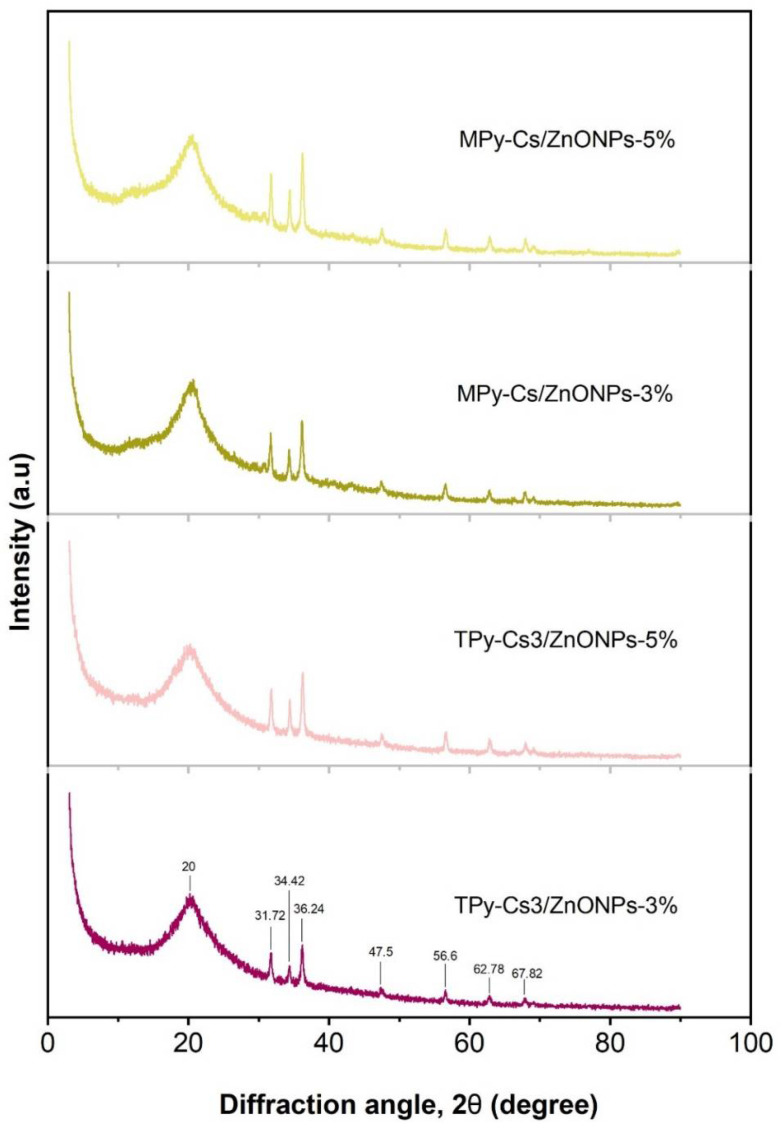
XRD patterns of MPy-Cs and TPy-Cs3-based ZnONP composites.

In order to confirm the incorporation of ZnONPs into the matrices of MPy-Cs and TPy-Cs, XRD measurements were performed (Figure 8). All the patterns of MPy-Cs/ZnONPs and TPy-Cs/ZnONP composites showed seven new peaks, beside the amorphous peak of MPy-Cs and TPy-Cs, near 2θ = 20°, at 2θ = 31.72°, 34.42°, 36.24°, 47.5°, 56.6°, 62.78° and 67.82°, which were indexed to crystal planes of (100), (002), (101), (102), (200), (201), and (202), respectively. These peak and their corresponding crystal planes are in good agreement with those for the pure ZnO according to the standard (JCPDS card no. 36-1451). The intensity of these peaks increased with increasing the ZnONP content in the composites. This evidences the successful preparation of the MPy-Cs/ZnONPs and TPy-Cs/ZnONP composites [47].

#### 3.2.3. SEM Analysis

**Figure 9:**
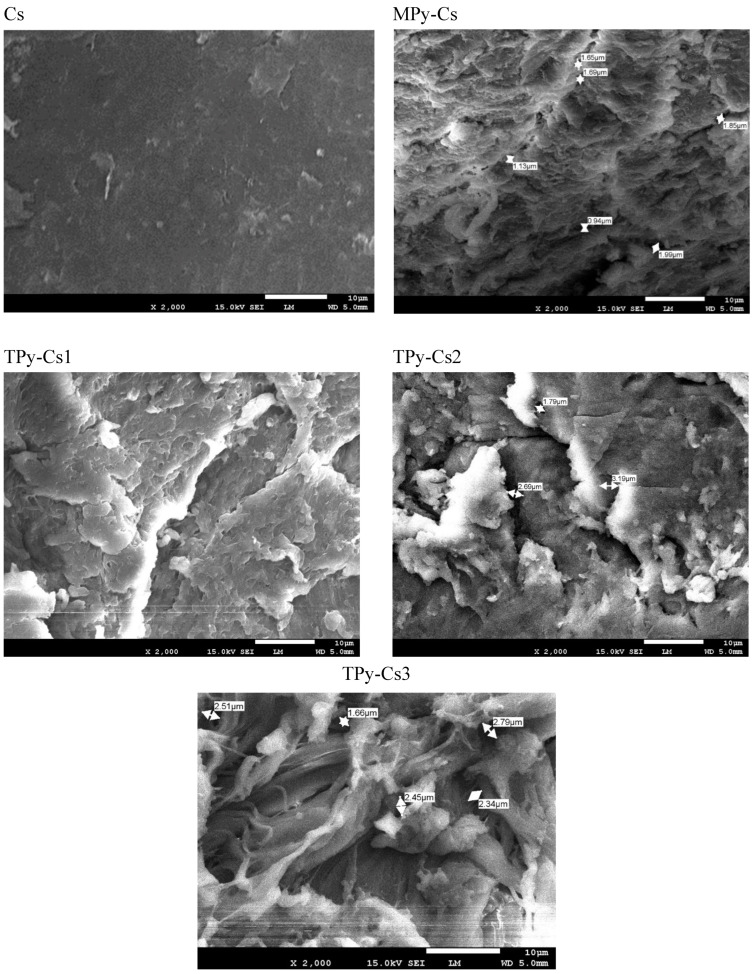
SEM images of MPy-Cs, and TPy-Cs derivatives.

Figure 9 depicts SEM images of topographical features on the surfaces of Cs, MPy-Cs, and TPy-Cs derivatives. The Cs surface was found to be smooth and free of any lumps. Upon cross-linking reaction, a drastic change in the surface appearance of chitosan has been observed. As compared with chitosan, the images confirm that the surfaces of its derivatives are rough and have grooves with a porous structure and high degree of lumps of varying sizes due to the differences in the size of the inserted modifier (MPy and TPy) by the crosslinking process. Further, the intensity of these lumps increased with increasing the crosslinking degree and can be arranged as follows: MPy-Cs = TPy-Cs1 > TPy-Cs2 > TPy-Cs3. It is assumed that their pores are considered to be the water penetration regions and the sites of interaction between the external stimuli and the hydrophilic functional groups of these derivatives. The incorporation of the long chain cross-linker MPy and TPy between the chitosan chains effectively reduces the hydrogen bonds between the chitosan chains resulting in the formation of the microporosity developing to a more open structure. These pores permit these derivatives to absorb a large quantity of water and polar solvents that support the interaction between them and the incoming ingredients. The images show that although the three TPy-Cs derivatives have a comparable surface appearance, and the distribution and the size of their pores are different from TPy-Cs1 to TPy-Cs3. Since the pores became denser with increasing the amount of the crosslinker from TPy-Cs3 to TPy-Cs1. Thus, these derivatives are characterized by a microporous structure with a large surface area which makes them able to be used as good drug delivery systems.

#### 3.2.4. TEM Analysis

**Figure 10:**
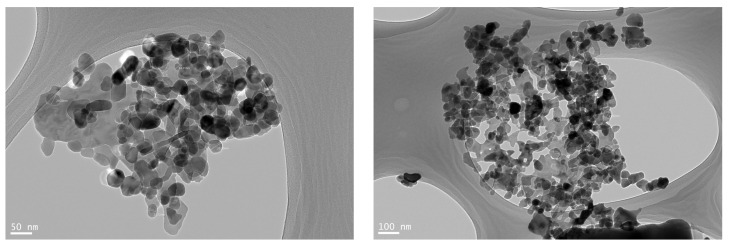
TEM images of TPy-Cs2/ZnONPs-5% composite at different magnifications.

TEM images of the TPy-Cs2/ZnONPs-5% composite, at different magnifications, are presented in Figure 10. The uniform dispersion of ZnONPs into the TPy-Cs2 matrix was clearly observed. The ZnONPs appeared as spherical-like shapes, and their sizes ranged from 14.21 to 29.20 nm.

### 3.3. Anticancer and Cytotoxic Activity

#### 3.3.1. Anticancer Activity

**Figure 11:**
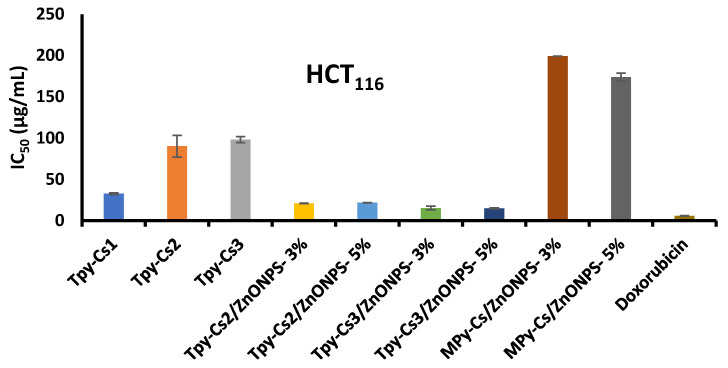
IC_50_ for the investigated MPy-Cs, TPy-Cs derivatives, and their ZnONP composites, beside DOX (as reference drug) against HCT_116_ cancer cell line at 48 h incubation. The results are expressed as the mean ± SD of three separate experiments performed in three replicates.

**Figure 12:**
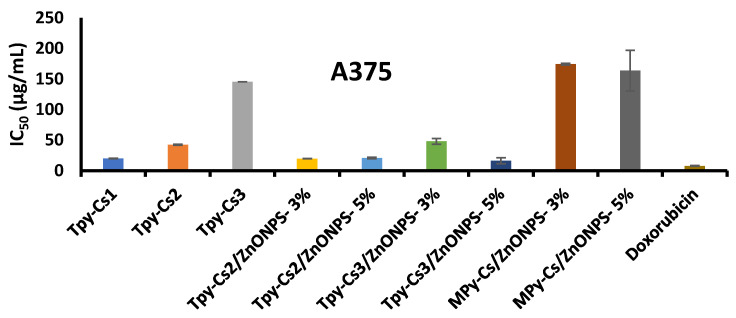
IC_50_ for the investigated MPy-Cs, TPy-Cs derivatives, and their ZnONP composites, beside DOX (as reference drug) against A375 cancer cell line at 48 h incubation. The results are expressed as the mean ± SD of three separate experiments performed in three replicates.

**Figure 13:**
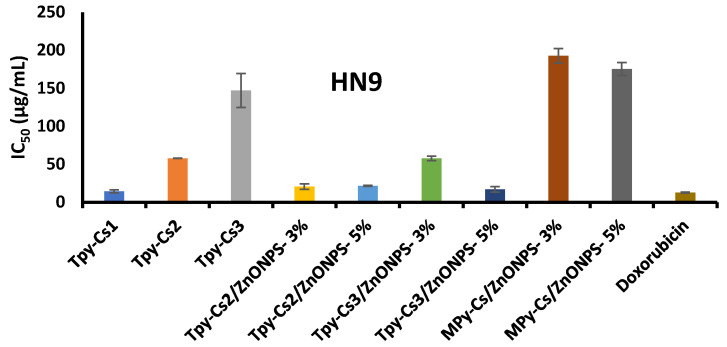
IC_50_ for the investigated MPy-Cs, TPy-Cs derivatives, and their ZnONP composites, beside DOX (as reference drug) against HN9 cancer cell line at 48 h incubation. The results are expressed as the mean ± SD of three separate experiments performed in three replicates.

The activity of the newly synthesized samples, including MPy-Cs, TPy-Cs derivatives, and their ZnONP composites against the three cancer (HCT_116_, A375, and HN9) cell lines using sulphorhodamine-B (SRB) assay was examined. Their ability to cause 50% inhibition of the cell growth (IC_50_) relative to the DOX reference drug is shown in Figure 11, Figure 12 and Figure 13 and Table 2. The cancer cells were treated with doses 25, 50, 100, and 200 μg/mL of the investigated samples solubilized in DMSO. The Half-maximal Inhibitory Concentration (IC_50_) value of each sample was calibrated from the graph of the dose–response curve for each concentration using Graph Pad Prizm software, (version 8.2). The complete data, from which IC_50_ was calculated, were provided via non-published material. In comparison to TPy-Cs derivatives, the MPy-Cs derivative exhibited no signs of activity against all the examined cancer cell lines, suggesting the role of thiocarbonyl group in enhancing the anticancer activity. This was supported by the activity of the TPy-Cs derivatives for inhibiting the tested cancer cell lines that greatly improved with increasing their thiopyrazole content, i.e., from TPy-Cs3 to TPy-Cs1. This was illustrated by their IC_50_ values against all the inspected cancer cell lines. Against the HCT_116_ cell line, the IC_50_ values of TPy-Cs3, TPy-Cs2, and TPy-Cs1 were 98.07, 90.12, and 32.64 μg/mL, respectively (Figure 11 and Table 2). Against the A375 cell line, the IC_50_ values of TPy-Cs3, TPy-Cs2, and TPy-Cs1 were 145.66, 42.53, and 20.10 μg/mL, respectively (Figure 12 and Table 2). Against the HN9 cell line, the IC_50_ values of TPy-Cs3, TPy-Cs2, and TPy-Cs1 were 147.19, 57.87, and 14.40 μg/mL, respectively (Figure 13 and Table 2).

In comparison to the activity of the reference Doxorubicin drug (IC_50_ = 12.60 μg/mL) against the HN9 cell line, derivative TPy-Cs1 (IC_50_ = 14.40 μg/mL) showed very close activity to that of this drug. On the other hand, it was found that the level of the anticancer activity of the other two TPy-Cs derivatives (TPy-Cs2 and TPy-Cs3) is greatly lower than that of the reference Doxorubicin drug; thus, they were selected for the creation of some ZnONP bio-composites to enhance their anticancer activity. As would be predicted, incorporating ZnONPs into the matrix of TPy-Cs2 appreciably enhanced its anticancer activity as evidenced by dropping its IC_50_ value from 90.12 to 20.98 and 21.68 μg/mL for TPy-Cs2/ZnONPs-3% and TPy-Cs2/ZnONPs-5%, respectively, against the HCT_116_ cell line (Figure 11 and Table 2). Also, against the A375 cell line, the IC_50_ value of TPy-Cs2 improved from 42.53 to 19.67 and 20.63 μg/mL for TPy-Cs2/ZnONPs-3% and TPy-Cs2/ZnONPs-5%, respectively (Figure 12 and Table 2). Similarly, against the HN9 cell line, the IC_50_ value of TPy-Cs2 lowered from 57.87 to 20.57 and 21.6 μg/mL for TPy-Cs2/ZnONPs-3% and TPy-Cs2/ZnONPs-5%, respectively (Figure 13 and Table 2).

**Figure 14:**
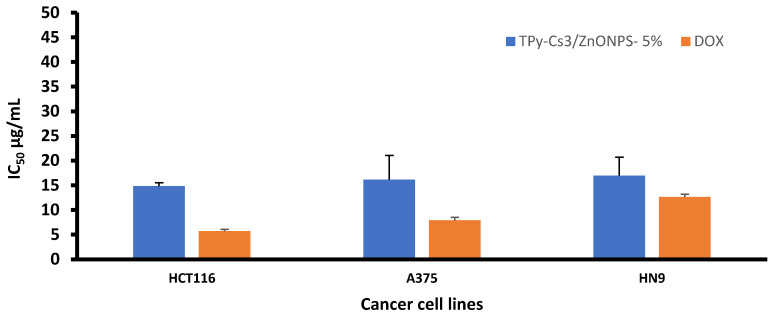
IC_50_ for TPy-Cs3/ZnONPs-5% against the three cancer cell lines at 48 h incubation. The results are expressed as the mean ± SD of three separate experiments performed in three replicates.

An analogous behavior was observed when ZnONPs were impregnated into the matrix of TPy-Cs3, since its IC_50_ value decreased from 98.07 to 15.21 and 14.82 μg/mL for TPy-Cs3/ZnONPs-3% and TPy-Cs3/ZnONPs-5%, respectively, against the HCT_116_ cell line (Figure 11 and Table 2). Again, its IC_50_ value reduced from 145.66 to 48.06 and 16.14 μg/mL for TPy-Cs3/ZnONPs-3% and TPy-Cs3/ZnONPs-5%, respectively, against A375 cell line (Figure 12 and Table 2). Also, its IC_50_ value decreased from 147.19 to 57.69 and 16.95 μg/mL for TPy-Cs3/ZnONPs-3% and TPy-Cs3/ZnONPs-5%, respectively, against the HN9 cell line (Figure 13 and Table 2). Thus, the TPy-Cs3/ZnONPs-5% was observed to be the most potent anticancer candidate against all the tested cell lines (Figure 14), although it does not exceed the anticancer activity of Doxorubicin.

Although the MPy-Cs derivative has no potency to inhibit the activity of the three inspected cancer cell lines, its ZnONP composites showed a moderate one. IC_50_ of MPy-Cs/ZnONPs-3% and MPy-Cs/ZnONPs-5% were 199.35 and 173.89 μg/mL, respectively, against the HCT_116_ cell line (Figure 11 and Table 2), 174.38 and 163.79 μg/mL, respectively, against the A375 cell line (Figure 12 and Table 2), 192.86 and 175.33 μg/mL, respectively, against the HN9 cell line (Figure 13 and Table 2).

#### 3.3.2. Cytotoxic Activity

**Figure 15:**
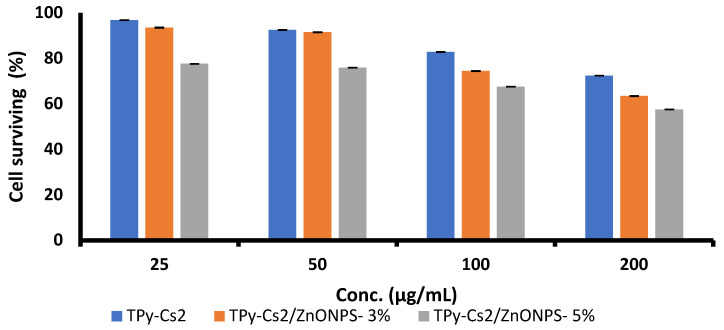
Cytotoxicity of TPy-Cs2 derivative and its ZnONP composites on the normal human skin fibroblast (HSF) cell line. The results are expressed as the mean ± SD of three separate experiments performed in three replicates.

The toxic action of the TPy-Cs2 derivative and its ZnONP composite (as a representative example of the prepared samples) on normal human skin fibroblast cell line (HSF) was inspected at a concentration range between 25 and 200 μg/mL (Figure 14 and Table 3). The results revealed that at a concentration of 25 μg/mL of the TPy-Cs2 derivative, TPy-Cs2/ZnONPs-3% and TPy-Cs2/ZnONPs-5% composites had a slight impact on the viability of the HSF cells (96.77, 93.55 and 77.67%, respectively), as can be observed in Figure 15 and Table 3. A progressive lowering in the viability was obtained by increasing the concentration of the investigated samples to 200 μg/mL. The viability of the HSF cells at 200 μg/mL concentration of TPy-Cs2 derivative, TPy-Cs2/ZnONPs-3% and TPy-Cs2/ZnONPs-5% composites was 72.44, 63.49 and 57.59%, respectively. Thus, the cytotoxicity for 50% of HSF cells (CC_50_) was found to be over 200 μg/mL concentration of the three inspected samples. As noted in Figure 10, Figure 11 and Figure 12 and Table 2, the TPy-Cs2 derivative and TPy-Cs2/ZnONPs-3% and TPy-Cs2/ZnONPs-5% composites demonstrated a better activity against all the inspected cancer cell lines at lower IC_50_ values (ranged from 20.63–90.12 μg/mL) than that which resulted during the assessment of the cytotoxicity (CC_50_ > 200 μg/mL). This illustrates the safe features of these samples and suggests that they can be used as one of the suitable anticancer drugs.

#### 3.3.3. Structure–Activity Relationship (SAR)

The promising anticancer properties of every component of the synthesized composites, including chitosan, pyrazole, and ZnONPs, prompted us to assess their inhibitory activity to gain insight into the mechanism of the anticancer activity of these composites with various concentrations.

The molecular insight into the anticancer activity of these targets involves a manifold approach, with membrane transport playing a critical role. These composites influence the increased permeability and retention (EPR) effect, which causes tumor tissues to accumulate preferentially. In cellular biology, the term “membrane transport” refers to a collection of mechanisms that control the passage of ions and other small molecules across biological membranes, which are made up of protein-containing lipid bilayers. Passage through the membrane is controlled by selective membrane permeability, a characteristic of biological membranes that enables them to segregate components of different chemical natures.

Drugs are delivered into cells via the influence of the attached function groups. In detail, the unique qualities of promising biopolymer chitosan facilitate cellular damage through interactions between its positively charged amino groups and the negatively charged cancer cell membranes and the hydroxyl-rich chitosan backbone facilitating electrostatic interactions with the negatively charged cell membrane. Furthermore, chitosan stimulates apoptosis pathways in cancer cells and suppresses angiogenesis, which is the formation of blood vessels that feed tumors. Additionally, its distinctive biocompatibility and biodegradability improve the targeted delivery of anticancer drugs to tumor cells precisely [48].

**Figure 16:**
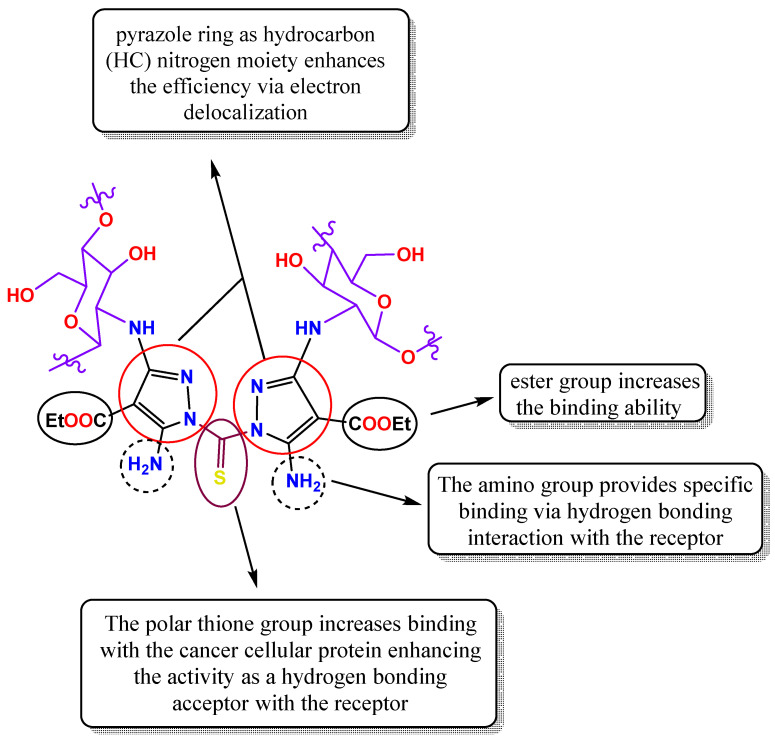
Structure–activity relationship for the TPy-Cs derivatives.

The introduction of substituted pyrazole ligand with its nitrogen-containing heterocycle (which is associated with amino and ester plus the important thione groups) contributes to the composite’s ability to intercalate with DNA, disrupting its structure and inhibiting replication via binding with the protein membrane (Figure 16). Additionally, the aforementioned IC_50_ values of the tested pyrazolo-chitosan derivatives affirmed the much greater inhibitory potential of the TPy-Cs derivatives compared to the MPy-Cs derivative. This may be due to the presence of thione (C=S) groups, which enhance their inhibition activity via improvement of the binding between the investigating TPy-Cs derivatives and the amino acid of cancer cell membrane lining protein, leading to the damage of the cancer cell [49].

On the other hand, the pyrazole moiety can coordinate with ZnONPs, influencing their release kinetics and stability. Numerous previous research studies have demonstrated that ZnONPs have therapeutic anticancer potential due to their unique features. This is due to a series of steps that occur in cancer cells, beginning with a loss of inner balance and redox state instability, followed by Zn^+^ created by oxidation of ZnONP internalization facilitated by endocytosis, followed by lysosomal escape, allowing the nanoparticles to be agglomerated in the cytoplasm and membranes of cells, which causes cellular damage and leads to cell death. Also, oxidative stress causes DNA damage, resulting in cell demise [50,51,52]. Therefore, the loading of 3% and 5% concentrations of ZnONPs into the synthesized pyrazole cross-linked chitosan derivatives played a vital role in the enhancement of the inhibitory activity against the employed cancer cell lines.

From the displayed results, it was concluded that the ideal concentration among all samples is 5% ZnONPs loaded into TPy-Cs3 (TPy-Cs3/ZnONPs-5%), as it exhibited the most potency against the three examined tumor cells.

The synergy between these functional groups—the cationic chitosan, the DNA-intercalating functionalized pyrazole moiety, and the redox-active ZnONPs—creates a versatile anticancer mechanism. This mechanism involves enhanced cellular uptake, membrane disruption, DNA damage, mitochondrial dysfunction, and cell cycle arrest, finally leading to apoptosis. The strategic combination of these functionalities provides a targeted and potent therapeutic effect, minimizing off-target toxicity and maximizing efficacy.

## 4. Conclusions

Some new pyrazole derivatives based on biopolymer chitosan were successfully produced. This was achieved through crosslinking of chitosan chains using either malonopyrazole to obtain MPy-Cs derivative or three different concentrations of thiopyrazole to obtain three TPy-Cs derivatives: TPy-Cs1, TPy-Cs2, and TPy-Cs3 with crosslinking degrees of 71, 48, and 29%, respectively. Moreover, impregnation of different amounts of ZnONPs into most of the prepared TPy-Cs and MPy-Cs derivatives was carried out using 3 and 5% ZnONPs based on the weight of these derivatives, attaining the corresponding ZnONP bio-composites. Some analytical techniques (FTIR, XRD, SEM, and TEM) were used to prove the chemical, inner, and morphological structure of these derivatives and their corresponding ZnONP composites. Their anticancer performance was significantly enhanced with an increase in their TPy and ZnONP content. Despite the fact that the MPy-Cs derivative showed no inhibition activity against all the inspected cancer cell lines, TPy-Cs derivatives possessed a considerable anticancer activity which was boosted as a function of their thiopyrazole content, i.e., from TPy-Cs3 to TPy-Cs1. Further, the TPy-Cs1 exhibited IC_50_ (14.4 μg/mL) against the HN9 cell line that was comparable to the standard drug Doxorubicin (12.6 μg/mL). Amongst all the obtained composites, TPy-Cs3/ZnONPs-5% has the most anticancer potency against all the checked cell lines, although it did not surpass the anticancer potency of Doxorubicin. The results indicated the safe features of Tpy-Cs2 and its ZnONP composites on normal human skin fibroblast (HSF) cell lines. Thus, the synthesized Tpy-Cs and their ZnONP composites can be used as one of the suitable anticancer drugs. So, it can be concluded that a combination of TPy, ZnONPs, and chitosan in the same formulation may be deemed as a good approach to improve the anticancer performance of the produced composites.

## Figures and Tables

**Table 1:** Synthesis, elemental analysis and crosslinking degree of novel TPy-Cs derivatives.

Derivative Code	MA-Cs: TCH (Molar Ratio)	MA-Cs (g)	TCH (g)	Elemental Analysis (%)					Crosslinking Degree (%)
				C	H	N	O	S	
Cs	-	-	-	44.79	6.82	8.65	39.74	-	-
TPy-Cs1	1: 0.5	2	0.32	44.80	5.09	16.60	28.79	4.72	71
TPy-Cs2	1: 0.25	2	0.16	44.80	5.66	13.99	32.38	3.17	48
TPy-Cs3	1: 0.125	2	0.08	44.79	6.12	11.88	35.29	1.92	29

**Table 2:** Anticancer activity for the tested MPy-Cs, TPy- Cs derivatives, and their ZnONP composites against HCT116, A375, and HN9 cell lines. The results are expressed as the mean ± SD of three separate experiments performed in three replicates.

Sample	HCT_116_		A375		HN9	
	IC_50_ (μg/mL)	SD (±)	IC_50_ (μg/mL)	SD (±)	IC_50_ (μg/mL)	SD (±)
TPy-Cs1	32.64	1.08	20.10	0.35	14.40	1.97
TPy-Cs2	90.12	13.21	42.53	0.82	57.87	0.16
TPy-Cs3	98.07	3.66	145.66	0	147.19	22.34
TPy-Cs2/ZnONPs-3%	20.98	0.33	19.67	0.28	20.57	3.52
TPy-Cs2/ZnONPs-5%	21.68	0.28	20.63	1.21	21.60	0.61
TPy-Cs3/ZnONPs-3%	15.21	2.29	48.06	4.59	57.69	2.89
TPy-Cs3/ZnONPs-5%	14.82	0.69	16.14	4.91	16.95	3.74
MPy-Cs	-	-	-	-	-	-
MPy-Cs/ZnONPs-3%	199.35	0	174.38	1.35	192.86	9.49
MPy-Cs/ZnONPs-5%	173.89	4.69	163.79	33.28	175.33	8.63
DOX	5.68	0.37	7.89	0.59	12.60	0.60

**Table 3:** Cytotoxic effect of TPy-Cs2 derivative and its ZnONP composites on the normal human skin fibroblast (HSF) cell line. The results are expressed as the mean ± SD of three separate experiments performed in three replicates.

Sample Conc. (μg/mL)	TPy-Cs2		TPy-Cs2/ZnONPs- 3%		TPy-Cs2/ZnONPs- 5%	
	Surviving (%)	SD (±)	Surviving (%)	SD (±)	Surviving (%)	SD (±)
0	100	0.00	100	0.00	100	0.00
25	96.77	0.04	93.55	0.04	77.67	0.04
50	92.57	0.04	91.55	0.07	75.98	0.01
100	82.84	0.02	74.49	0.05	67.59	0.03
200	72.44	0.04	63.49	0.03	57.59	0.01

## Data Availability

The data presented in this study are available on request from the corresponding authors.
